# Rapid Reduction of Pro-Inflammatory Cytokines with an Oral Topical Composition Comprising Olive Oil, Trimethylglycine and Xylitol: A Randomized Double-Blind Controlled Trial

**DOI:** 10.3390/ijms26104920

**Published:** 2025-05-21

**Authors:** José López-López, José M. Reuss, Teresa Vinuesa-Aumedes, Sonia Egido-Moreno, Xavier Roselló-Llabres, Tanya Pereira-Riveros, Debora Reuss, Laura Alonso-Gamo, Beatriz Rodríguez-Vilaboa

**Affiliations:** 1 Department of Odontostomatology, Faculty of Medicine and Health Sciences (Dentistry), University of Barcelona, 08907 Barcelona, Spain; 2 Medical Surgical Area, Dental Hospital University Barcelona (HOUB), 08907 Barcelona, Spain; 3 Oral Health and Masticatory System Group, Bellvitge Institute of Biomedical Research (IDIBELL), 08908 Barcelona, Spain; 4 Department of Postgraduate Prosthodontics, Faculty of Dentistry, Complutense University of Madrid, 28040 Madrid, Spain; 5 Unit of Microbiology, Department of Pathology and Experimental Therapeutics, Faculty of Medicine and Health Sciences, University of Barcelona, 08907 Barcelona, Spain; 6 Department of Odontostomatology, Faculty of Medicine, San Pablo CEU University, 28668 Madrid, Spain; 7 Department of Endocrinology, Paediatrics Unit, University Hospital Quirón Madrid—European University, 28223 Madrid, Spain; 8 Center for Microbiome and Inflammatory Science (CMIS), 28001 Madrid, Spain; 9 Clínica Vilaboa, 28001 Madrid, Spain

**Keywords:** inflammation, cytokines, IL-1β, TNF-α, overweight, obesity, microbiome dysbiosis, chronic inflammatory diseases, olive oil, trimethylglycine and xylitol, mucosa barrier disruption

## Abstract

An underlying pro-inflammatory status is related to recurrence and persistence of inflammatory susceptibility in obesity and periodontitis, two of the most prevalent chronic inflammatory diseases. Elevated levels of interleukin-1β (IL-1β) and tumor necrosis factor-α (TNF-α), part of the inflammatory network linking these two conditions, persist even after periodontal treatment, with high salivary cytokine levels being linked to overweight and obesity risk. This trial assessed the effect of a novel composition comprising olive oil, trimethylglycine and xylitol, delivered topically to the oral mucosa, on salivary cytokines in periodontally healthy normal and overweight/pre-obese individuals. In a randomized placebo-controlled double-blind clinical trial, adult patients were randomly assigned to use a test toothpaste (intervention group, IG) or a placebo toothpaste (control group, CG) three times a day for 1 month. Primary outcomes were levels of salivary cytokines IL-1β, TNF-α and interleukin-4 (IL-4). Significant differences between IG and CG were observed for IL-1β (*p* = 0.003; Z = 2.901; r = 0.62) and TNF-α (*p* = 0.001; Z = 3.23; r = 0.69), but not for IL-4 (*p* = 0.203; Z = 1.321; r = 0.28). A significant reduction in IL-1β (*p* = 0.008) and a near significant reduction in TNF-α (*p* = 0.059) was found in the IG at the end of the trial. Additionally, the effect of body mass index on cytokine levels response was analyzed. A significantly different behavior was shown between IG and CG in the overweight/pre-obesity subgroup for IL-1β (*p* = 0.014; Z = 2.430; r = 0.63) and TNF-α (*p* = 0.029; Z = 2.199; r = 0.57). Moreover, a significant decrease in IL-1β in the IG (*p* = 0.028) was observed. The rapid reduction in IL-1β and TNF-α after 1 month of use of the intervention composition suggests a safe and effective novel strategy for reducing pro-inflammatory cytokines that may offer an opportunity to diminish the inflammatory status in patients with overweight/pre-obesity.

## 1. Introduction

Inflammation understood as an acute self-limiting biological response of human immune system can be initiated by a variety of danger signals coming from either pathogens, injured and damaged cells, free radicals, poisons or exposure to toxic substances. Low-grade chronic inflammation refers to the persistent pro-inflammatory status where inflammation is not resolved or deficiently resolved.

Maintained external pressure on mucosa and skin barriers from environmental circumstances such as diet, life style and stress provides a landscape that paves the way to a shift from homeostasis to a disrupted host immune response. Sustained pressure can exceed immune system control mechanisms, resulting in an ungoverned release of a variety of pro-inflammatory cytokines such as interleukin-1β (IL-1β) and tumor necrosis factor-α (TNF-α) that further contributes to barrier breakage and to a state of immune dysregulation and inflammation. Scientific evidence shows that a pro-inflammatory cytokine overexpression leads to what is known today as metabolic infection and systemic inflammation [1].

Significant evidence supports the role of IL-1ß, TNF-α and interleukin-6 (IL-6) in chronic inflammation. These cytokines, produced mainly by macrophages and helper T cells, are generated in a cascade manner with one cytokine stimulating further release of other cytokines in a redundant pro-inflammatory action [2].

Interleukin-4 (IL-4) has been described both as anti-inflammatory and pro-inflammatory [3]. IL-6 has been linked mainly with insulin resistance and diabetes in obesity-derived chronic inflammation. Recently, a cooperative interaction between IL-1ß and TNF-α in regulating IL-6 expression in adipocytes has been demonstrated, both in lean and obese adipose tissues. However, this alliance is shown to be much higher in obese patients, contributing to the understanding of the high inflammatory burden in obesity [4].

In obesity, an expanded inflammatory status characterized by an increased level of cytokines IL-1ß and TNF-α generated by adipocyte macrophages is released to the blood and reaches periodontal tissues, among others, where more pro-inflammatory cytokines are secreted by macrophages located at the mucosa barrier [5]. This further contributes to deepen the dysbiotic shift, with additional release of cytokines, vindicated today as core to the inflammatory memory and comorbidities pathways [6].

The list of stressors and moderators of salivary markers of inflammation are continuously updated, with stress and body mass index (BMI) having received increasing attention. A higher BMI has been linked to higher levels of circulating inflammatory markers, such as IL-1β. Interestingly, when both stress and BMI are taken together, a higher BMI is associated with greater salivary IL-1ß reactivity to stress [7].

IL-1β and TNF-α, appointed as master pro-inflammatory cytokines, enter saliva through the blood flow, with a close relation between salivary and serum concentration. Passive diffusion and active transport from blood to saliva and vice versa takes place at the salivary gland tissue. This bidirectional pathway has already been evidenced linking local oral inflammation from periodontal origin with a manifold of chronic inflammatory diseases [8].

Periodontal inflammation, the second most frequent contributor to systemic inflammatory load behind obesity, is another important factor upregulating salivary pro-inflammatory cytokines, offering a practical model to address the complex crosstalk between inflammation and chronic inflammatory diseases [9].

Elevated levels of IL-1ß and TNF-α are key players in chronic inflammatory diseases such as obesity, diabetes, rheumatoid arthritis and inflammatory bowel disease, among others, as well as periodontitis [10,11]. Furthermore, elevated levels of IL-1ß and TNF-α have been also associated with neurodegenerative diseases such as Alzheimer’s disease and Parkinson’s disease [12,13]. Scientific evidence shows that the dysregulated secretion of IL-1ß, IL-6 and TNF-α is behind what is known today as neuroinflammation that directly exacerbates neuronal damage [14]. IL-1ß and TNF-α have also been implicated in cancer [15,16].

Moreover, IL-1ß is involved in the deleterious production of pro-inflammatory inducible nitric oxide (iNO) and reactive oxygen species (ROS) responsible for DNA damage, depriving the individual of anti-inflammatory activity towards a nascent tumor outgrowth. In fact, the implication of IL-1ß in inflammation-induced carcinogenesis is not new [16].

Additionally, IL-1β and TNF-α have shown to enhance bacterial growth in vitro, an effect that is inhibited in the presence of cytokine-neutralizing antibodies [17].

Pro-inflammatory cytokine reduction is already the target of new antibodies, biologicals and anti-IL-1ß and anti-TNF protocols, with important adverse effects, however [18]. The treatment of inflammatory comorbidities is nowadays directed onto systemic neutralization of cytokines with the appraisal of new therapies to neutralize IL-1ß in diseases traditionally approached in a symptomatic management such as the treatment of cardiovascular conditions [19].

Taking into account that global figures of chronic inflammatory diseases are continuously rising, it is essential to find innovative, safe and effective strategies to reduce pro-inflammatory cytokine release.

A composition comprising olive oil, trimethylglycine and xylitol topically delivered to the oral mucosa as a daily toothpaste for 4 months has shown a significant improvement of determinants of oral inflammation contributing to oral eubiosis in patients with gingivitis [20]. The effect on cytokine levels was not assessed in the above-mentioned study.

In this context, the present randomized controlled trial (RCT) aimed to assess the effect of the topical administration on the oral mucosa of said composition delivered via a daily toothpaste on salivary cytokines IL-1ß, TNF-α and IL-4 in periodontally healthy normal and overweight/pre-obesity patients as the primary objective. The secondary objective was to determine the effect of said composition on total subgingival bacterial load, bleeding on probing (BOP), and plaque index. Additionally, whether body mass index influences cytokine level response to the assigned intervention was assessed as an exploratory outcome. Lastly, the overall acceptability of the intervention product based on patient-reported outcomes was also determined.

## 2. Results

### 2.1. Demographic and Clinical Characteristics of Participants

**Figure 1:**
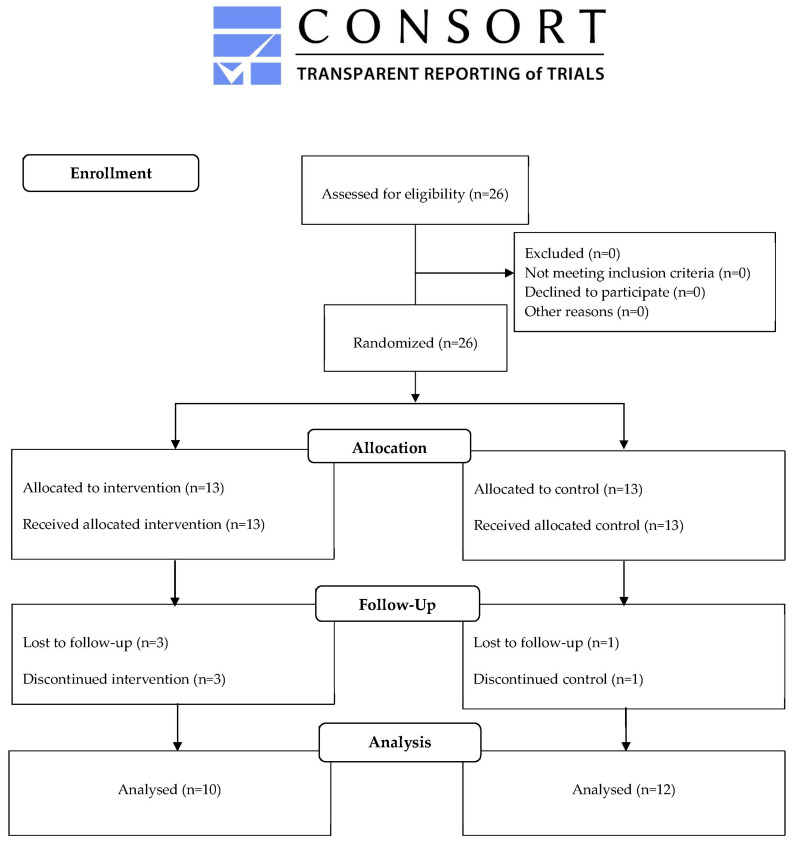
CONSORT 2010 flow diagram of participants through the trial.

This trial took place from June 2022 to September 2022. Patients were recruited during a routine visit to University of Barcelona Dental Hospital. A total of 26 participants were assessed for eligibility and enrolled in the study. Four patients did not attend the second visit and were thus excluded from the trial. From the remaining 22 patients, 10 belonged to the intervention group (IG) and 12 to the control group (CG) (Figure 1).

At baseline, both groups were similar in terms of age and sex, although a non-significant higher percentage of women was observed in the control group. Both groups were also homogeneous in terms of body mass index. No significant differences were observed in IL-1ß, TNF-α, IL-4 and total bacterial load between groups at the beginning of the study (Table 1).

### 2.2. Inflammatory Cytokine Levels in Total Population

Median concentration of inflammatory cytokine markers before and after treatment for each group (IG and CG) is shown in Table 2.

A significant reduction in IL-1ß (*p* = 0.008) was observed in the intervention group, whereas a significant increase in TNF-α levels (*p* = 0.01) was found in the control group after 4 weeks of treatment. A near significant decrease in TNF-α levels (*p* = 0.059) was found in the intervention group, while in the control group a non-significant elevation of IL-1ß (*p* = 0.21) was seen after 1 month of treatment. No significant changes were observed for IL-4 neither in the intervention nor control groups (Table 2).

In the intergroup analysis, significant differences were found for IL-1ß (*p* = 0.003; Z = 2.901; r = 0.62) and TNF-α (*p* = 0.001; Z = 3.23; r = 0.69), whereas a non-significant difference was found for IL-4 (*p* = 0.203; Z = 1.321; r = 0.28).

Analysis of combined data from the total population, that is, IG plus CG (n = 22 participants), showed no significant changes in cytokine levels or bacterial loads after the trial, ruling out any general effect of trial participation or improved health behaviors in the interpretation of the results (Supplementary Table S1).

### 2.3. Inflammatory Cytokine Levels in Overweight/Pre-Obesity Subgroup

A significant different behavior was shown both for IL-1β (*p* = 0.014; Z = 2.430; r = 0.63) and TNF-α (*p* = 0.029; Z = 2.199; r = 0.57) between intervention and control groups in the overweight/pre-obese subgroup. Whereas a reduction in both IL-1β and TNF-α was observed in the overweight/pre-obesity population in the intervention group, an increase in both cytokines was found in the overweight/pre-obesity population in the control group. Additionally, a significant intragroup reduction in IL-1β levels was observed in the overweight/pre-obesity population in the intervention group (*p* = 0.028) (Table 3). By contrast, no intergroup nor intragroup significant differences were observed in the normoweight subgroup, neither in the IG nor CG (Supplementary Table S2).

The analysis of cytokine levels in the combined data from the total population attending to BMI, that is, normoweight plus overweight/pre-obesity (n = 21 participants), showed no significant variations in cytokine levels by the end of the trial, ruling out general effects of trial participation or improved health behaviors in lifestyle along the duration of the study (Supplementary Table S3).

### 2.4. Secondary Outcomes

Regarding total bacterial load, a non-significant different behavior was seen between the two groups (*p* = 0.16). While the intervention reduced bacterial counts (*p* = 0.51), the control group showed an increase in bacterial load (*p* = 0.29) (Table 4).

Intergroup analysis showed non-significant differences in BOP, plaque index and salivary pH after 1 month. No significant changes were found regarding salivary flow (Supplementary Tables S4 and S5).

### 2.5. Adverse Effects and Overall Acceptability of Intervention Composition

No intolerance or adverse effects were reported neither in the intervention nor in the control group. In the intervention group, 80% of the participants reported a feeling of better oral health, as well as rated the flavor of the toothpaste above 9 in the visual analogue scale (VAS) 0–10. In addition, 90% resulted in score ≥ 8 to the question whether they would use/recommend the product.

## 3. Discussion

This randomized controlled double-blind clinical trial has shown a significant decrease in IL-1ß levels (*p* = 0.008), with a near significant reduction in TNF-α (*p* = 0.059) after 1 month of use of a novel composition comprising olive oil, trimethylglycine and xylitol, delivered through a daily toothpaste.

The nine-fold decrease in IL-1β (median of 29.14 pg/mL) and the almost three-fold reduction in TNF-α levels (median of 2.07 pg/mL) observed in the intervention group at the end of the treatment resulted in values that were in the range of those reported for healthy individuals [24,25]. When comparing the pro-inflammatory cytokine changes observed between intervention and control groups, a significant difference was found for IL-1ß (*p* = 0.003; Z = 2.901; r = 0.62) and for TNF-α (*p* = 0.001; Z = 3.23; r = 0.69).

In the current RCT, initial median values of IL-1β in intervention (median of 263.48 pg/mL) and control groups (median of 294.65 pg/mL) were in the range reported for periodontal inflammation [26]. Similarly, TNF-α initial median values were in the range of those reported for periodontal inflammation, both in the intervention and control groups (median of 5.27 pg/mL and 3.15 pg/mL, respectively), despite the fact that participants were periodontally healthy patients with bleeding index < 10% compatible with health [27].

It has been reported that although salivary IL-1β levels in patients with periodontitis were significantly reduced after non-surgical periodontal therapy, they still remained significantly higher than in healthy controls [28]. Additionally, recent research has evidenced oral microbiome dysbiosis persistence after completion of dental treatment and clinical disease remission, suggesting this could be a possible underlying mechanism for disease recurrence [29].

It is noteworthy that oral dysbiosis in obese patients remains even after weight loss, and it is found to be pivotal to the recently coined obesogenic memory, upregulating inflammatory cytokines and NLRP3 inflammasome, and actively enhancing the release of IL-1ß and TNF-α, among others [30,31]. These pro-inflammatory cytokines have been said to become then epicenters of the inflammatory response.

It is accepted nowadays that a low-grade inflammation is activated early during adipose tissue expansion such as during the transition from normal to overweight, while in higher BMI, such as in obesity, a permanent pro-inflammatory phenotype is thought to be fundamental in the progression to a high burden of disease [32]. Significantly higher levels of pro-inflammatory cytokines have been observed in overweight/pre-obesity and obesity patients with BMI ≥ 25 [33].

Taking into account that obesity is nowadays the chronic inflammatory disease exerting the highest inflammatory burden, we decided to evaluate the effect of the intervention in regards to BMI. In the current trial, at baseline, there were no significant differences regarding BMI between groups, with 71% being overweight/pre-obesity patients (BMI 25–29.9) and 29% being of normal weight (BMI < 25).

The significant IL-1β reduction found in the intervention group was also observed when analyzing the subgroup of participants with BMI ≥ 25. A significant different response to the assigned intervention in the overweight/pre-obese patients is to be exclusively attributable to the specific intervention itself, as the analysis of cytokine levels in the total population attending to BMI (normoweight and overweight participants) at the end of the trial showed no significant variations, thus ruling out any effects of trial participation or improved health behaviors.

In the current trial, a trend to increase total subgingival bacterial load was seen in the control group, while a trend to decrease total bacterial load was found in the intervention group. This effect could be related to the reduction in IL-1β and TNF-α observed in the current trial, in accordance with the literature reporting the bidirectional effect between proinflammatory cytokines and bacterial overgrowth [17,34].

Along the 1-month duration of the current trial, no significant changes in oral health variables such as BOP, plaque index and salivary flow were found neither between groups nor within the groups. However, significant improvement in gingival bleeding has been previously reported in gingivitis patients with higher baseline BOP levels (in the range of 44 ± 18%) using the tested toothpaste for a longer period of time (4 months) [20]. This could be due to the low level of BOP at baseline or the shorter duration of the present study.

Another explanation would be that the rapid reduction in the inflammatory cytokines found in the current trial shows a potential biological activity that precedes the improvement of any other health parameters.

Interestingly, one prospective study in rheumatoid arthritis (RA) and periodontitis patients exposed to Etanercept, an anti-TNF-α drug, showed a 1.21-fold reduction in TNF-α in crevicular fluid after 6 weeks, with no periodontal intervention in the meantime, and a significant improvement in gingival index and BOP [35].

In a controlled trial performed in patients with RA and chronic periodontitis (CP) (RA+CP group), CP and healthy subjects (control), TNF-α levels in crevicular fluid only showed a 1.47-fold reduction after 6 weeks of receiving periodontal therapy in patients with RA+CP, but not in patients with RA [36].

On the other hand, in an extensive systematic review and metanalysis of RCT studying the effect of dietary weight loss intervention on IL-6 and TNF-α in adults with obesity, only IL-6 was reported to be significantly reduced after at least 5% of weight loss. However, TNF-α remained unchanged in dietary weight loss interventions [37].

Relevantly, the rapid reduction in pro-inflammatory cytokines after 1 month found in the current trial differs from the delayed reduction in IL-1β, TNF-α and IL-6 that happens after 6 months in bariatric and metabolic surgery patients [38]. Furthermore, in a recent clinical trial evaluating the effect of glucagon-like peptide 1 receptor agonists (GLP-1RA) administration in a group of patients with type 2 diabetes and dyslipidemia, a significant reduction in plasmatic IL-1β and TNF-α levels was observed after 6 months of treatment with either semaglutide or dulaglutide [39]. Interestingly, the magnitude of cytokine reduction reported in the above-mentioned study (1.26-fold for IL-1β and 1.38-fold for TNF-α) was notably lower than that observed in the current trial after 1-month use of the tested toothpaste (nine-fold for IL-1β and 2.55-fold for TNF-α).

Mounting evidence supports the involvement of IL-1β, IL-6 and TNF-α in the mechanisms of pathological pain, which is another aspect of inflammation [2]. Pain was not analyzed in the current trial as it was an exclusion criterion.

The tested composition has been evaluated in a yet unpublished phase 3 RCT (NCT05463484), where a metagenomic approach was performed to assess its effect on oral microbiota and other health related pathways such as nitric oxide (NO) pathway.

A limitation of the current trial is the small sample size since there have been patients lost to follow-up in the size initially considered. Nevertheless, the reduction in pro-inflammatory cytokine levels observed in the intervention group compared to the control group was statistically significant (*p* < 0.05) when considering both the total population (n = 22) and the overweight/pre-obesity subgroup (n = 15). Additionally, the calculated effect sizes indicated a strong effect of the intervention composition (r > 0.50; Table 2 and Table 3), demonstrating its impact regardless of sample size. Further interventional studies with larger sample size are being performed.

## 4. Materials and Methods

### 4.1. Study Design and Participants

The current study was designed as a randomized placebo-controlled double-blind clinical trial in adult patients scheduled for a routine visit at the University of Barcelona (UB), Dental Hospital Barcelona University (HOUB), Bellvitge Campus. The study protocol was approved by the Ethics Committee of the UB Dental Hospital with ethical approval number 20/2022 strictly following the ethical principles established in the Declaration of Helsinki for medical investigations performed in human beings as established by the World Medical Association, October 2013. Each participant signed an informed consent form prior to the initiation of the study.

Participation in the trial was voluntary, respecting the principle of autonomy. Patients were informed of the nature and purpose of the study.

Privacy and data security of participants were protected at all times in accordance with the provisions of Organic Law 3/2018. Patients were able to withdraw from the study at any time without the need to provide justification.

Inclusion criteria were: Patients 18 years and older attending a routine dental visit to the university clinic, reporting no pain neither infection at the time of the visit, being medically stable individuals classified as ASA I (healthy with no systemic diseases) or ASA II (had mild, well-controlled medical conditions that did not interfere with their daily activities) according to the American Society of Anesthesiologists (ASA), patients not exceeding BMI of 40 kg/m^2^, periodontally healthy or with mild periodontitis.

Exclusion criteria were: Patients not willing to participate, allergic to any components of the composition of the tested and control toothpastes, patients under antibiotic treatment or patients having had antibiotics during the last 7 days, patients with any type of orthodontic treatment, such as brackets or others, patients with diabetes, BMI > 40 kg/m^2^, and severe periodontitis.

This study followed the Consolidated Standards of Reporting Trials (CONSORT) reporting guidelines [40]. The CONSORT Flow diagram with study enrolment, visits, and attrition is shown in Figure 1. This trial is registered in Clinical Trials.gov (NCT06786910).

### 4.2. Randomization and Masking

Once consented, patients were randomly assigned by means of a random assignment sequence generated using Microsoft Office Excel 2019 (Microsoft Corporation, Washington, DC, USA, 2013). A restricted allocation with a 1:1 distribution was used to assign participants to either the Intervention Group (IG) or the Control Group (CG).

Intervention group participants were given an experimental toothpaste containing a novel intellectually protected antiseptic-free composition comprising olive oil, trimethylglycine and xylitol. The control group was to use a placebo toothpaste with an identical aspect, flavor and ingredients as the intervention toothpaste, except for it did not comprise said composition.

All products were packaged in white tubes to allow for masking and blinding within the study. The specific product information allocated to each patient was placed in a sealed envelope, exhibiting exclusively patient allocation number to maintain the integrity of the blinding process. Participants, care providers and researchers were masked to assignment. Randomization and allocation were performed by trained staff members at HOUB.

### 4.3. Procedures

Participants from both groups were to be seen at the dental clinic on two occasions. First visit included baseline data collection and assignment to either group, IG or CG. Second and final visit was scheduled after 1 month (30 ± 5 days) for final data collection.

- First Visit (T0):

Sex and age were recorded from medical history. Clinicians performed extra and intraoral examinations. A new orthopantomography was taken if the existing one was over a year old.

No periodontal treatment including scaling and root planning nor oral hygiene instruction was performed before or during the study period. Participants were asked to maintain their usual cleaning routine, such as brushing, flossing or the use of interdental brushes.

Throughout the trial, patients were instructed to refrain from using any additional chemical agents for dental hygiene, nor to use any other toothpaste. No mouth rinsing was to be performed by the participants either.

The following data were recorded: oral mucosa examination; decayed, missing and filled teeth (DMFT) index [41]; O’Leary plaque index [42]; basic periodontal examination (BPE; probing depth, supra or subgingival calculus/overhangs, bleeding on probing (BOP), and recessions and clinical attachment loss (CAL), if present) [22,23]; sialometry (unstimulated and stimulated salivary flow) [43]; salivary pH [44]; subgingival total bacterial load; and salivary cytokine levels (Supplementary Materials and Methods).

Microbiological analysis: Samples were taken with sterile paper points inserted into the gingival sulcus of at least three teeth, preferably from 16, 22, 41, 47 (if present in the mouth, otherwise the closest medial tooth). Impregnated paper points were placed in a 2 mL cryotube and sent immediately to the microbiology laboratory of UB Bellvitge Hospital, where they were frozen at −80 °C for further processing by real-time quantitative polymerase chain reaction (qPCR) using TaqMan assay. Total bacteria load, expressed as logarithmic units (log) of colony forming units per mL (cfu/mL), was determined by amplifying 16S rRNA gene using universal procariotic primers (Supplementary Materials and Methods).

Cytokine analysis: Samples from unstimulated saliva were sent immediately to the microbiology laboratory of UB Bellvitge Hospital. They were centrifuged (14,000 rpm, 10 min, 4 °C), and supernatants were stored at −80 °C until determination of TNF-α, IL-1β, and IL-4 concentrations by enzyme linked immunosorbent assay (ELISA) using commercial kits (BioLegend ELISA MaxDeluxe Kit Human TNF, IL-1β and IL-4). Plates were read with a Multiskan Ms Labsystems microplate reader (Thermo Fisher Scientific, Altrincham, UK).

Unstimulated and stimulated saliva were collected after 1 h of fasting. Salivary flow was measured and expressed in mL/min. Data were categorized as Normal, Low or Very Low salivary flow [43]. For determining unstimulated salivary flow, patients tilted their head forward to collect saliva into a glass along 5 min. Saliva volume was then measured in a graduated tube. For determining stimulated salivary flow, patients in the same described position chewed paraffin for 5 min. Saliva was then collected into a glass over the following 5 min and subsequently measured in a graduated tube.

Participant weight and height were recovered. Body mass index (BMI) was calculated as weight in kilograms divided by the square of height in meters (kg/m^2^), and participants were classified according to their nutritional status as underweight (< 18.5), normal weight (BMI: 18.5–24.9), overweight/pre-obese (BMI: 25–29.9) and obese (BMI ≥ 30) [21] (Supplementary Table S6).

At the end of the visit, patients received their randomized assigned toothpaste, instructed to brush their teeth three times a day and refrain from using any other oral hygiene products. The second and final visit was scheduled.

II. Second Visit (T1):

The procedure from first visit was repeated, collecting the same data. Additionally, patients were requested to rate ease of use, flavor, acceptance, preference and satisfaction/recommendation to use of the assigned toothpaste via a visual analogue scale (0 to 10). At this time, oral prophylaxis was performed in due patients.

### 4.4. Outcomes

The primary outcomes of the study were the levels of inflammatory cytokines (IL-1ß, TNF-α, and IL-4) in saliva samples at baseline and after 1 month of use of interventional and of placebo composition.

Secondary outcomes were: total bacteria load, bleeding on probing and plaque index at baseline and after 1 month of use of interventional and of placebo composition.

An exploratory outcome of the trial was the effect of body mass index on cytokine level response to the assigned intervention. Another outcome evaluated was the patient- reported outcome measure in relation to ease of use, flavor, acceptance, preference and satisfaction/recommendation to use.

Participants were asked to report whether they experienced any adverse side effects, such as staining, change in flavor, oral mucosa dryness, allergic reactions, swelling, sensitivity, discomfort and/or pain.

### 4.5. Statistical Analysis

Sample size was calculated using α = 0.05, ß = 0.2 and a two-tailed significance level of 95%, applying the Student’s t-test for independent samples. A standardized difference of d = 1.1 on Cohen’s scale was assumed for comparing inflammatory cytokine levels between groups. Since salivary cytokines are significantly correlated to bleeding on probing [26], BOP was used as a proxy for inflammation. Data from a previous study using the same composition [20] provided the BOP values for this estimate. Based on this, a minimum of 13 patients per group was required.

Data were analyzed with the SPSS Statistics software version 26. Categorical variables were described in frequency and percentage. Numerical variables, according to their distribution, in mean and standard deviation or median and interquartile range were expressed. Categorical variables were compared using the chi-square test for sex and t-student test for age. Numerical variables under study (inflammatory markers and bacterial load), having a non-normal distribution, were compared using non-parametric tests. To determine the differences of values between the two groups (intervention and control) at the two times evaluated (T0: baseline; T1: after 1-month treatment) the Mann–Whitney U test was used for independent samples. To determine intragroup differences before and after the intervention/control, Wilcoxon’s signed range test was used for related samples. A *p* < 0.05 value was considered statistically significant.

For analyzing whether there were intragroup differences in salivary flow, Fisher’s test for independent samples and the McNemar test for related samples were used.

Regarding effect size calculation for primary outcomes, since the variables did not follow a normal distribution, non-parametric tests were used. The effect size, represented as r-value, was calculated using the absolute value of the Z statistic obtained from the Wilcoxon or Mann–Whitney test, divided by the square root of the sample size. For their interpretation, the following r-values were used: 0.01–0.29 represents small effect sizes, 0.30–0.49 medium effect sizes and 0.50–1.00 large effect sizes [45].

## 5. Conclusions

The main results of the current RCT demonstrate that the use of a novel composition comprising olive oil, trimethylglycine and xylitol in a toothpaste for 4 weeks was able to reduce salivary pro-inflammatory cytokines IL-1β and TNF-α towards health levels. The rapid and strong reduction in pro-inflammatory cytokines observed after just one-month intervention cannot be attributable to any periodontal intervention nor hygiene regimen implementation different from patient’s previous routine, highlighting the potential of the oral mucosa-delivered composition in the downregulation of an inflammatory status. Therefore, the intervention composition constitutes a promising safe and effective strategy for diminishing pro-inflammatory cytokines IL-1β and TNF-α in chronic inflammatory conditions, such as overweight/pre-obesity.

## Figures and Tables

**Table 1:** Baseline characteristics of participants.

Variables	Intervention (n = 10)	Control (n = 12)	*p*-Value
Age (years old), mean (SD)	43.8 (16.7)	53.2 (24.7)	0.32
Gender, n (%)			
Female	5 (50%)	10 (83.3%)	0.09
Male	5 (50%)	2 (16.7%)	
Anthropometric parameters, n (%) *^#^			
Normal weight (BMI ≤ 24.9)	2 (22.2%)	4 (33.3%)	0.66
Overweight/Pre-obesity (25 ≤ BMI ≤ 29.9)	7 (77.8%)	8 (66.7%)	
Obese (BMI ≥ 30)	0	0	
Basic Periodontal Examination (BPE), n (%) ^†^			
Code 1	4 (40%)	4 (33.3%)	0.949
Code 2	3 (30%)	4 (33.3%)	
Code 3	3 (30%)	4 (33.3%)	
DMFT, mean (SD) ^‡^	13.6 (9.2)	13.8 (6.4)	0.628
Inflammatory mediators (pg/mL), median (IQR)			
IL-1ß	263.48 (124.74–563.01)	294.65 (78.85–513.82)	0.87
TNF-α	5.27 (1.20–14.38)	3.15 (2.40–4.26)	0.28
IL-4	12.53 (7.99–26.37)	0.25 (0.0–22.01)	0.09
Total subgingival bacterial load (log cfu/mL), median (IQR)	5.48 (3.39–6.46)	4.18 (1.81–5.19)	0.15

Data are mean (standard deviation, SD), n (%) or median (interquartile range, IQR). * Body Mass Index (BMI, kg/m^2^) as defined by WHO (2000) [21]; ^#^ n = 21. One out of the total participants (n = 22) did not declare height and weight data. ^†^ According to Palmer and Floyd (2023) [22] and Dietrich et al. (2019) [23]. ^‡^ Decayed, missing and filled teeth. IL-1β, interleukin-1β; TNF-α, tumor necrosis factor-α; IL-4, interleukin-4; log cfu/mL, logarithmic units of colony forming units per mL.

**Table 2:** Inflammatory cytokine markers at baseline and after 1-month treatment in the intervention group and control group.

Inflammatory Cytokine Levels (pg/mL)	Intervention (n = 10)	Control (n = 12)	*p*-Value (Between Groups)	r-Value (Effect Size)
IL-1 ß				
T0	263.48 (124.74–563.01)	294.65 (78.85–513.82)		
T1	29.14 (0.0–263.22)	355.77 (110.76–570.96)		
Difference (T1-T0)	−160.06 (−326.29–(54.29))	66.17 (−71.40–139.99)	0.003	Z = 2.901 r = 0.62
*p*-value (intra-group)	0.008	0.21		
TNF-α				
T0	5.27 (1.20–14.38)	3.15 (2.40–4.26)		
T1	2.07 (0.21–5.07)	7.73 (2.96–28.38)		
Difference (T1-T0)	−3.44 (−9.90–(-1.03))	5.00 (0.04–20.97)	0.001	Z = 3.23 r = 0.69
*p*-value (intra-group)	0.059	0.01		
IL-4				
T0	12.53 (7.99–26.37)	0.25 (0.0–22.01)		
T1	15.61 (5.05–23.62)	9.85 (0.0–28.98)		
Difference (T1-T0)	−3.77 (−14.28–10.66)	3.17 (-0.37–12.27)	0.203	Z = 1.321 r = 0.28
*p*-value (intra-group)	0.57	0.21		

Data are median (IQR). T0 = First visit. T1 = Second visit.

**Table 3:** Inflammatory cytokine markers in the overweight/pre-obesity subgroup at baseline and after 1-month treatment in the intervention and control groups.

Inflammatory Cytokine Levels (pg/mL)	Overweight/Pre-Obesity (n = 15)		*p*-Value (Between Groups)	r-Value (Effect Size)
	Intervention (n = 7)	Control (n = 8)		
IL-1 ß				
T0	169.79 (60.00–551.67)	316.66 (113.86–486.80)		
T1	0.00 (0.00–109.14)	454.41 (145.23–564.74)		
Difference (T1-T0)	−140.06 (−350.18–(−37.18))	82.73 (−36.61–210.97)	0.014	Z = 2.430 r = 0.63
*p*-value (intra-group)	0.028	0.208		
TNF-α				
T0	5.94 (0–26.90)	2.90 (2.07–4.26)		
T1	2.34 (0.24–4.40)	7.16 (1.96–19.65)		
Difference (T1-T0)	−3.92 (−22.50–0.30)	3.33 (−0.29–16.17)	0.029	Z = 2.199 r = 0.57
*p*-value (intra-group)	0.176	0.128		
IL-4				
T0	9.87 (4.20–25.88)	0.35 (0.00–21.81)		
T1	14.41 (6.73–24.20)	18.61 (0.00–28.62)		
Difference (T1-T0)	−3.13 (−14.27–20.00)	5.68 (−4.88–20.60)	0.613	Z = 0.579 r = 0.15
*p*-value (intra-group)	1.000	0.249		

Data are median (IQR). T0 = First visit. T1 = Second visit.

**Table 4:** Bacterial concentration at baseline and after 1-month treatment in the intervention group and control group.

Total Bacterial Count (log cfu/mL)	Intervention (n = 10)	Control (n = 11)	*p*-Value (Between Groups)
T0	5.48 (3.39–6.46)	4.18 (1.81–5.19)	
T1	4.81 (2.51–5.69)	4.45 (1.98–6.82)	
Difference (T1-T0)	−1.20 (−2.10–1.79)	1.14 (−1.28–2.46)	0.16
*p*-value (intra-group)	0.51	0.29	

Data are median (IQR). T0 = First visit. T1 = Second visit. Log cfu/mL, logarithmic units of colony forming units per mL.

## Data Availability

Data are contained within the article or Supplementary Materials. The original contributions presented in this study are included in the article/Supplementary Materials. Further inquiries can be directed to the corresponding author.

## Supplementary Materials

The following supporting information can be downloaded at https://www.mdpi.com/article/10.3390/ijms26104920/s1.

## Abbreviations

The following abbreviations are used in this manuscript:

BMI	body mass index
BOP	bleeding on probing
CAL	clinical attachment loss
CFU	colony forming units
CG	control group
BPE	basic periodontal examination
DMFT	decayed, missing and filled teeth
ELISA	enzyme linked immunosorbent assay
GLP-1RA	glucagon-like peptide 1 receptor agonists
IG	intervention group
IL-1β	interleukin-1β
IL-4	interleukin-4
IL-6	interleukin-6
i-NO	inducible nitric oxide
NLRP3	nucleotide-binding domain and leucine-rich repeat pyrin containing protein-3
NO	nitric oxide
qPCR	quantitative polymerase chain reaction
RCT	randomized controlled trial
ROS	reactive oxygen species
TNF-α	tumor necrosis factor-α
VAS	visual analogue scale
