# Supplementary material for: Rapid Reduction of Pro-Inflammatory Cytokines with an Oral Topical Composition Comprising Olive Oil, Trimethylglycine and Xylitol: A Randomized Double-Blind Controlled Trial
Source: Int J Mol Sci. 2025 May 21;26(10):4920. doi: 10.3390/ijms26104920 (PMC12112298; doi:10.3390/ijms26104920)
Supplement: Supplementary file 1 [file ijms-26-04920-s001.zip › ijms-3603810-supplementary.pdf]

Supplementary Materials

Table S1. Levels of inflammatory cytokines and total bacterial load before and after treatment in total population (IG+CG).

**Inflammatory cytokines (pg/mL) and bacterial load (log cfu/mL)**	**Total population (n = 22)**	***p*-value (intra-group)**
IL-1 ß		
T0	263.45 (99.88-543.53)	
T1	155.85 (29.43-441.53)	
Difference (T1-T0)	-48.60 (-184.65-91.80)	0.27
TNF-α		
T0	4.05 (2.13-5.95)	
T1	4.65 (1.28-11.08)	
Difference (T1-T0)	0.10 (-3.25-8.90)	0.57
IL-4		
T0	9.60 (0-25.23)	
T1	13.50 (0-25.50)	
Difference (T1-T0)	0.0 (-10.03-11.70)	0.63
Total subgingival bacterial load		
T0	4.45 (2.43-5.75)	
T1	4.45 (2.45-6.31)	
Difference (T1-T0)	-1.02 (-1.51-1.82)	0.74

Data are median (interquartile range, IQR). IG = intervention group. CG = control group. T0 = First visit. T1 = Second visit. IL-1β, interleukin-1β; TNF-α, tumor necrosis factor-α; IL-4, interleukin-4; log cfu/ml, logarithmic units of colony forming units per mL.

Table S2. Inflammatory cytokine markers in the normoweight subgroup at baseline and after 1-month treatment in the intervention and control group.

Inflammatory cytokine levels (pg/ml)	Normoweight (n=6)		*p*-value (between groups)
	**Intervention (n=2)**	**Control (n=4)**	
IL-1 ß			
T0	497.52 (395.02-600.03)	248.47 (55.20-471.92)	
T1	356.97 (312.23-401.72)	204.83 (102.74-461.92)	
Difference (T1-T0)	-140.55 (-198.31-(-82.79))	25.98 (-67.57-105.11)	0.267
p-value (intra-group)	0.180	0.715	
TNF-α			
T0	7.40 (4.61-10.20)	3.79 (2.85-14.98)	
T1	4.43 (1.81-7.06)	22.33 (5.92-43.02)	
Difference (T1-T0)	-2.97 (-3.14-(-2.80))	12.58 (2.69-28.43)	0.133
p-value (intra-group)	0.180	0.068	
IL-4			
T0	26.43 (25.02-27.84)	0.25 (0.00-12.16)	
T1	22.85 (22.28-23.42)	3.57 (0.00-18.65)	
Difference (T1-T0)	-3.58 (-4.41-(-2.74))	3.17 (-0.25-6.73)	0.133
p-value (intra-group)	0.180	0.285	

Data are median (IQR). T0 = First visit. T1 = Second visit.

Table S3. Levels of inflammatory cytokines before and after treatment in normoweight and overweight/pre-obesity total population* (IG+CG).

Inflammatory cytokines (pg/mL)	Normoweight (n = 6)	Overweight/Pre-obesity (n = 15)	*p*-value (between groups)
IL-1β			
T0	390.78 (110.39-557.30)	202.75 (68.33-540.84)	
T1	297.61 (126.67-401.72)	109.14 (0.00-480.26)	
Difference (T1-T0)	-57.19 (-103.55-83.55)	-37.18 (-235.49-116.67)	0.970
p-value (intra-group)	0.600	0.510	
TNF-α			
T0	4.39 (3.40-10.20)	3.92 (1.45-5.94)	
T1	6.56 (5.79-38.60)	3.8 (0.30-9.40)	
Difference (T1-T0)	2.69 (-2.80-21.66)	0.00 (-3.92-7.40)	0.302
p-value (intra-group)	0.249	0.925	
IL-4			
T0	12.16 (0.00-25.02)	9.25 (0.00-25.88)	
T1	14.71 (0.00-23.42)	14.41 (0.00-27.91)	
Difference (T1-T0)	-0.25 (-2.74-6.33)	0.00 (-10.74-20.00)	0.733
p-value (intra-group)	0.686	0.422	

Data are median (IQR). *n = 21. One out of the total participants (n = 22) did not declare height and weight data. IG = intervention group. CG = control group. T0 = First visit. T1 = Second visit.

Table S4. Plaque index and bleeding on probing at baseline and after 1-month treatment in the intervention and control group.

**Intra-oral parameters**	**Intervention (n = 10)**	**Control (n = 12)**	***p*-value (between groups)**
O'Leary plaque index (%)			
T0	37.20 (21.42-70.45)	36.28 (21.30-77.80)	
T1	38.00 (24.12-62.40)	36.75 (22.36-76.75)	
Difference (T1-T0)	-1.22 (-3.89-0.23)	0.32 (-2.02-1.63)	0.346
p-value (intra-group)	0.241	0.754	
Bleeding on probing (%)			
T0	3.84 (2.46-12.17)	7.49 (4.62-13.21)	
T1	3.11 (0.80-8.90)	6.74 (4.15-9.71)	
Difference (T1-T0)	-0.39 (-1.66-0.33)	-0.55 (-0.99-0.41)	0.923
p-value (intra-group)	0.241	0.158	

Data are median (IQR). T0 = First visit. T1 = Second visit.

Table S5. Other variables analysed at baseline and after 1-month treatment in the intervention and control group.

Variables		T0 (n = 22)		T1 (n = 22)	
		**Intervention (n = 10)**	**Control (n = 12)**	**Intervention (n = 10)**	**Control (n = 12)**
pH, mean (SD)		6.6 (0.5)	6.9 (0.5)	6.8 (0.3)	6.9 (0.6)
Unstimulated salivary flow*	N	6	7	8	7
	L	3	4	2	4
	VL	1	1	0	1
Stimulated salivary flow*	N	7	8	8	8
	L	2	3	2	2
	VL	1	1	0	2

Data are mean (standard deviation, SD) or *number of participants presenting N, L or VL salivary flow. N = Normal. L = Low. VL = Very low. T0 = First visit. T1 = Second visit.

Table S6. Nutritional status for adults over 20 years old according to World Health Organization [21].

**Body mass index (BMI)***	**Nutritional status**
Below 18.5	Underweight
18.5–24.9	Normal weight
25.0–29.9	Pre-obesity
30.0–34.9	Obesity class I
35.0–39.9	Obesity class II
Above 40	Obesity class III

*BMI is expressed in kg/m^2.

Supplementary Materials and Methods

- DMFT index: Number of decayed (D), missing (M), and filled teeth (F) [41].

- O´Leary Plaque Index: Percentage of stained surfaces (mesial, distal, buccal, palatal/lingual) out of the total number of tooth surfaces [42].

- Basic Periodontal Examination (BPE): Probing depth, supra or subgingival calcules/overhangs, bleeding on probing (BOP). Additionally, recessions and clinical attachment loss (CAL) were measured, when present [22, 23].

Probing depth and CAL at 6 sites per tooth were measured using the CP-12 probe. BPE divides the full dentition into sextants. The six sextants include, on one hand, four groups of teeth comprising the molars (excluding the third molar) and premolars on each side of each jaw (sextants 1 and 6 on the right side and sextants 3 and 4 on the left side). On the other hand, there are two groups of teeth comprising the canines and incisors of each jaw (sextants 2 and 5). All teeth in each sextant are examined. The results are recorded in a chart:

1	2	3
6	5	4

BPE Code:

**Code 0**	no periodontal pocket depth ≥ 4 mm; no dental calculi nor overhangs; no BOP.
**Code 1**	no periodontal pocket depth ≥ 4 mm; no dental calculi nor overhangs; BOP.
**Code 2**	no periodontal pockets depth ≥ 4 mm; dental calculi or overhangs; BOP.
**Code 3**	periodontal pockets depth between 4-5.5 mm; BOP.
**Code 4**	periodontal pockets depth > 6 mm; BOP.
**Code ***	furcation involvement

- Sialometry: Unstimulated and stimulated salivary flow were measured and expressed in mL/min. Data were categorized as Normal, Low or Very Low salivary flow [43].

Unstimulated salivary flow (mL/min)	Category
> 0.25	Normal
0.10-0.25	Low
< 0.10	Very low

Stimulated salivary flow (mL/min)	Category
> 1.0	Normal
0.7-1.0	Low
<0.7	Very low

• Salivary pH: Salivary pH was measured using FILTERLAB paper strips (WHA10360005, Whatman® Panpeha™ pH indicator strips, Barcelona, Spain) ranging from 1 to 14, and following manufacturer instructions [44].

• Microbiological analysis: Primers and probes used for determining total bacteria by q-PCR (LineGene 4800 thermocycler, Bioer Technology, Hangzhou, China) were the following: forward:5´-TCCTACGGGAGGCAGCAGT--3´; reverse: 5´-GGACTACCAGGGTATCTAATCCTGTT--3`; probe: 5´-6FAM-CGTATTACCGCGGCTGCTGGCAC-TAMRA-3`). PCR conditions were: initial amplification cycle at 95º C for 10 min (denaturation), followed by 40 cycles at 95ºC for 15 seconds and 60º for 1 minute (extension). The TaqMan probe-based q-PCR approach used allowed quantification through the detection of fluorescence emitted at each amplification cycle, which was directly proportional to the accumulation of amplicons. The first cycle in which the detected fluorescence intensity surpassed the background signal was defined as the threshold cycle (Ct). Using specialized software, Ct values obtained from unknown samples were compared with those from positive controls containing known concentrations of target DNA, and from which equivalent log cfu/mL were calculated. These controls were derived from previously established standard curves, and an additional five-point calibration curve was included in each run to ensure accurate quantification.
